# Wound management for post-laryngeal transplant pharyngeal fistula: a case report

**DOI:** 10.1016/j.bjorl.2024.101439

**Published:** 2024-05-02

**Authors:** Jiahui Fan, Zheng Jiang, Mailudan Ainiwaer, Rong Yu, Dengying Gu, Huiling Zhao

**Affiliations:** a Department of Otolaryngology-Head and Neck Surgery, West China Hospital, Sichuan University/West China School of Nursing, Sichuan University, Sichuan Province, Chengdu, 610041, China; b Department of Otolaryngology-Head and Neck Surgery, West China Hospital, Sichuan University, Sichuan Province, Chengdu, 610041, China

## Introduction

Laryngeal cancer is one of the most common malignant cancers of the head and neck. Presently, primary modalities in laryngeal carcinoma treatment encompass surgical intervention, chemotherapeutic administration, radiotherapeutic application, and immunomodulatory strategies, each fraught with attendant drawbacks. Many patients with advanced laryngeal cancer undergo total laryngectomy, resulting in the loss of the larynx. In such cases, the functional capacity of the laryngeal region is compromised. The detriment to laryngeal functionality instigates a discernible diminishment in life quality, exerting pronounced ramifications upon communication, respiration, and deglutition processes. For individuals who have undergone complete laryngectomy, the transplantation of the laryngeal complex presently represents the exclusive therapeutic avenue facilitating the realization of these aspirations.^1^ Our institution has recently performed a successful heterograft larynx-trachea-thyroid transplantation ensuing the three documented instances of successful laryngeal transplantation worldwide. During the perioperative care, we encountered various challenges, among which, the wound infection was the most prominent Therefore, infection prevention and wound healing promotion are crucial postoperative care measures for laryngeal transplant procedures. In this report, we present a case of pharyngeal fistula occurring after laryngeal transplantation. Through extensive wound care, the pharyngeal fistula wound had completely healed within 7 days.

## Case report

A 65-year-old male patient, diagnosed with laryngeal carcinoma nine years ago, previously underwent a partial laryngectomy. In the current year, a recurrent neoplasm emerged, necessitating a total laryngectomy. The patient is disinclined toward this recourse due to apprehensions about vocal functionality forfeiture. Notwithstanding multiple instances of articulating the inclination for tumor extirpation coupled with preservation of laryngeal capabilities, protracted discourse with the surgical cohort culminated in the patient's solicitation for a laryngeal transplantation. On April 29, 2023, the patient underwent a narrow-field total laryngectomy under general anesthesia, with concomitant heterograft larynx-trachea-thyroid complex transplantation, in conjunction with bilateral reconstruction of the recurrent laryngeal and superior laryngeal nerves. On postoperative day 6 sputum analysis uncovered the presence of Carbapenem-Resistant Acinetobacter Baumannii (CRAB). On the seventh postoperative day, the patient exhibited modest serosanguinous discharge from the left cervical wound. Which was successfully managed with the combine use of various antibiotics and strengthen the dressing change for the cervical wound. By the thirteenth day, scanty exudation from the left cervical incision became perceptible, further manifested by fluid egress upon water ingestion ‒ a hallmark indicative of postoperative pharyngocutaneous fistulization. As a remedial measure, the metallic tracheostomy tube was replaced with a disposable cuffed endotracheal tube. Dressing regimen frequency of the cervical wound was escalated, predicated on evaluative monitoring of wound exudates. A specialized drainage tube was strategically emplaced at the fistulous juncture, concurrently applying continuous negative pressure within the range of 20–30 mPa. A therapeutic antibiotic transition ensued, involving the shift from intravenously administered piperacillin-tazobactam to a regimen encompassing vancomycin and meropenem, with supplementary integration of polymyxin B contingent upon culture findings sourced from the pharyngocutaneous fistula exudate. Marked resolution of the left cervical pharyngocutaneous fistula was healed on the twentieth day subsequent to the surgical intervention.

## Discussions

**Figure 1:**
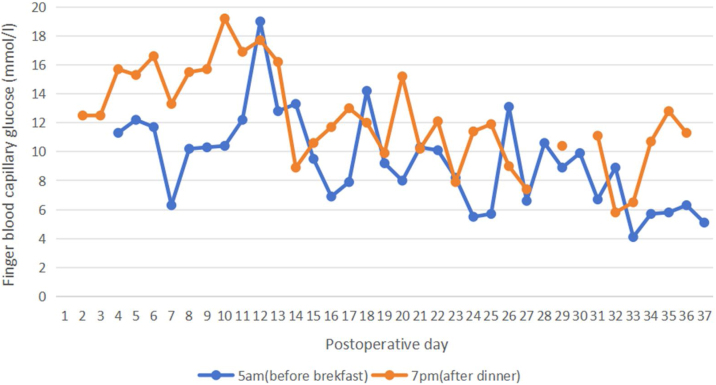
Postoperative trends in fingertip blood capillary glucose levels.

In typical circumstances, the immune system manages its relationship with the microbiota in a manner similar to its response to potentially harmful organisms. The use of immunosuppressive drugs after laryngeal transplantation presents an intriguing scenario. While these medications prevent the rejection of transplanted tissue, they concurrently weaken the patient's immune system, facilitating the colonization of harmful bacteria in the throat and the potential for infection. Additionally, immunosuppressive agents are known to impede the wound healing process.^2^ To preempt post-surgery rejection reactions, a corticosteroid treatment plan was devised through interdisciplinary collaboration. This plan involved the intravenous administration of methylprednisolone sodium succinate, with a dose of 500 mg on the day of surgery and 200 mg on the following day. Subsequently, the dosage was gradually reduced from 40 mg to 20 mg per day over a 24-day period, maintaining 20 mg daily until the day before discharge, the detailed methylprednisolone dosage was provided in Table 1. This pulsatile steroid treatment led to elevated blood glucose levels and affected the healing of the surgical connection in the neck,^3^ the changes of blood glucose level during the hospitalization was provided in Fig. 1. Furthermore, corticosteroids in isolation have been shown to hinder the wound healing process at various stages, including inflammation, wound strength, wound contracture, and epithelialization.^2^ Additionally, to facilitate the patient's early post-surgery swallowing exercises, the patient was advised not to use medications that reduce saliva production. Regrettably, this decision resulted in oral secretions adhering to the wound due to the accumulation of bacteria and enzymes, further elevating the risk of post-surgery infections.

**Table 1:** Dosage and Administration Method of methylprednisolone post-operation.

**Methylprednisolone Dosage**	200 mg	180 mg	140 mg	100 mg	60 mg	20 mg	20 mg	300 mg	20 mg
**Administration Method**	ivgtt	ivgtt	ivgtt	ivgtt	ivgtt	ivgtt	T.F	ivgtt	T.F
**Frequency**	**qd**								
Day post-surgery	1–2	3	4	5	6	7‒8	9‒10	11‒13	14‒37

Continuous negative pressure drainage is suitable for patients with relatively small fistulas (diameter < 3 cm).^4^ In this instance of laryngeal transplantation, the patient's pharyngocutaneous fistula measured approximately 1 cm in diameter. Employing the approach of continuous negative pressure drainage, the patient's fistula had fully healed by the 13th day. The occurrence of the pharyngocutaneous fistula prolonged the patient's hospitalization duration and increased associated costs. Therefore, the prevention of pharyngocutaneous fistula occurrence assumes even greater significance. Antibacterial agents based on silver (Ag) exhibit enduring bactericidal properties characterized by robust stability, a wide-ranging effectiveness against diverse bacterial strains, minimal vaporization propensity, and a reduced inclination to provoke the development of bacterial resistance.^5^ In this case of laryngeal transplantation, the post-operative risk of wound infection is notably elevated. To proactively prevent post-operative wound infection and the occurrence of pharyngocutaneous fistulas, the early application of antimicrobial dressings such as silver dressings can be integrated into the care of the patient's neck incision site.

## Conclusion

we presented a case of post-laryngeal transplantation with the complication of pharyngocutaneous fistula. The transplantation procedure involved a class II incision, and the application of immunosuppressive agents and glucocorticoids contributed to an increased risk of post-operative wound infection. Continuous Negative Pressure Wound Therapy as an effective therapeutic approach for addressing post-laryngeal transplant pharyngocutaneous fistula. Whether early utilization antimicrobial dressings can be employed to preempt the occurrence of wound infection warrants attention and further discussion.

## Declaration

On behalf of all the authors, I, corresponding author, confirm that all listed authors met the authorship criteria and that all authors are in agreement with the content of the manuscript.

## Authors’ contributions

The authors (JF, JZ, HZ) designed the study and wrote the manuscript. The authors (JF, MA, RY, JZ, HZ) performed the study and collected the data. All authors (JF, MA, RY, JZ, HZ) read and approved the final version for submission.

## Funding

This study is supported by Sichuan Province Cadre Health Research Project (2024-119). The funders had no role in the study design, data collection and analysis, decision to publish, or preparation of the manuscript.

## Conflicts of interest

The authors declare no conflicts of interest.
